# Lavender for Hospitals

**Published:** 1923-08

**Authors:** Winifred M. Lee.


### CORRESPONDENCE.

### Lavender for Hospitals.

*To the Editor of* THE HOSPITAL AND HEALTH REVIEW.

Sir,—Having helped to collect lavender for the sick and wounded during the war, and found it was greatly appreciated, it occurred to me that many people who have lavender bushes in their gardens would be only too willing to give it for use in ordinary hospitals. If any of your readers would be so kind as to send me dried lavender (shredded, if possible) I will have it made up into lavender bags and sent to various hospitals, chiefly in large towns, where a refreshing breath of the country is so welcome to the sick in the heat of summer, and also to naval and military hospitals. Verbena is also very much liked.

My address till the end of August will be Chapel House Farm Hotel, Chipping Norton.

WINIFRED M. LEE.

